# COVID-19, Suffering and Palliative Care: A Review

**DOI:** 10.1177/10499091211046233

**Published:** 2021-09-16

**Authors:** Tan Seng Beng, Carol Lai Cheng Kim, Chai Chee Shee, Diana Ng Leh Ching, Tan Jiunn Liang, Mehul Kumar Narendra Kumar, Ng Chong Guan, Lim Poh Khuen, Lam Chee Loong, Loh Ee Chin, Sheriza Izwa Zainuddin, David Paul Capelle, Ang Chui Munn, Lim Kah Yen, Nik Nathasha Hani Nik Isahak

**Affiliations:** 1 Department of Medicine, Faculty of Medicine, University of Malaya, Kuala Lumpur, Malaysia; 2 Department of Medicine, Faculty of Medicine and Health Science, University Sarawak Malaysia, Sarawak, Malaysia; 3 Department of Psychological Medicine, Faculty of Medicine, University of Malaya, Kuala Lumpur, Malaysia

**Keywords:** COVID-19, suffering, palliative care, symptom control, communication, team care

## Abstract

According to the WHO guideline, palliative care is an integral component of COVID-19 management. The relief of physical symptoms and the provision of psychosocial support should be practiced by all healthcare workers caring for COVID-19 patients. In this review, we aim to provide a simple outline on COVID-19, suffering in COVID-19, and the role of palliative care in COVID-19. We also introduce 3 principles of palliative care that can serve as a guide for all healthcare workers caring for COVID-19 patients, which are (1) good symptom control, (2) open and sensitive communication, and (3) caring for the whole team. The pandemic has brought immense suffering, fear and death to people everywhere. The knowledge, skills and experiences from palliative care could be used to relieve the suffering of COVID-19 patients.

## Introduction

**Figure 1.**
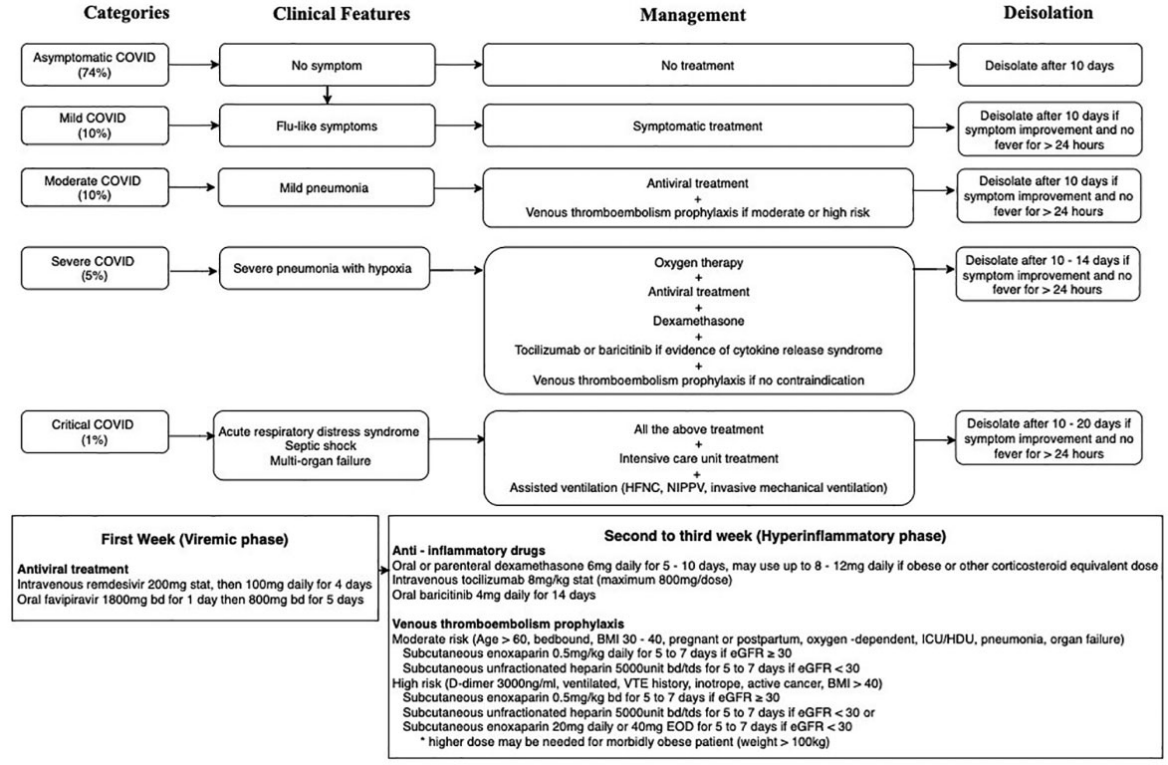
Covid 19 at a glance.

**Figure 2.**
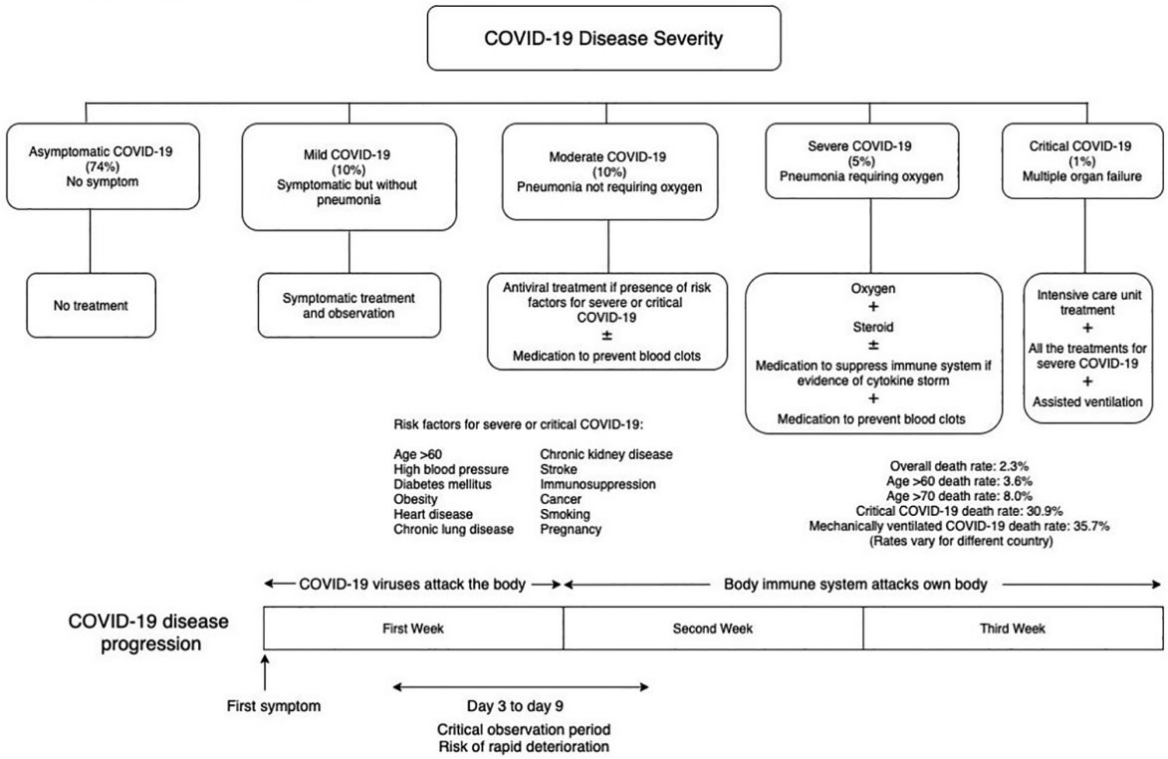
Medical information for Covid 19 patients and their family members at a glance.

Palliative care is the active holistic care of individuals with health-related suffering due to severe illness.^1^ It aims to relieve suffering and improve quality of life.^2^ The need of palliative care is not limited to cancer and chronic diseases, but also to those who are critically ill, including patients with severe COVID-19.^3-6^ In this review, we aim to provide a simple outline on COVID-19, suffering in COVID-19, and the role of palliative care in COVID-19. We also present 3 summaries: COVID-19 in Figure 1, COVID-19 patient information in Figure 2, and COVID-19 and palliative care in Table 1.

**Table 1.** COVID-19 and Palliative Care at a Glance.

Good symptom control		
Dyspnea	Non-pharmacological	Cool wipes, menthol lozenges, cool room temperature, avoid fan due to potential aerosol generation, loose clothing, prone positioning, forward lean position, near-window bed, body scan exercise, 20-minute mindful breathing
	Pharmacological	Oxygen therapy, corticosteroids, dihydrocodeine 15 mg 30 min before exertion for exertional dyspnea, dihydrocodeine 15-30 mg tds for resting dyspnea, promethazine 25-50 mg tds ±50 mg on, syrup morphine 2.5 mg prn/q4h, mirtazapine 15 mg on
	End-of-life dyspnea	SC morphine 2.5 mg prn/q1h (no renal failure) CSCI morphine 0.5 mg/h (no renal failure + multiple dosing required) SC fentanyl 10 mcg prn/q1h (renal failure) CSCI fentanyl 10 mcg/h (renal failure + multiple dosing required) SC midazolam 2.5 mg prn/q1h (agitation) CSCI midazolam 0.5 mg/h (agitation + multiple dosing required)
Cough	Non-pharmacological	Treat underlying causes, identify and avoid cough triggers (cold air, cold drinks, dry atmospheres, certain food and spices, exertion, talking), drink warm water, honey, mindful coughing (surf the urge and huff if necessary); for productive cough—huffing, incentive spirometry, self-administered chest physiotherapy LEGA if fit
	Pharmacological	Codeine 15-30 mg prn/qid, syrup morphine 2.5 mg prn/q4h, tiotropium inhaler 18 mcg daily, gabapentin 300 mg on-tds (max 600 mg tds), pregabalin 150 mg bd, N-acetylcysteine 200 mg tds (max 600 mg bd)
Fever	Non-pharmacological	Rehydration, cool wipes, reducing room temperature, consume cold drinks or ice-cream, loose clothing, light bedding
	Pharmacological	Paracetamol 1 g prn/qid, ibuprofen 200-400 mg prn/qid, CSCI diclofenac sodium 150 mg over 24 hours for dying patients with fever
Anxiety Depression Sleep disturbances	Non-pharmacological	Anxiety and depression–relaxation exercises, breathing exercises, online psychological interventions Insomnia–treat nocturnal symptoms, avoid steroids and diuretics after 2 pm, increase daytime activity, limit daytime sleep, limit fluid intake in the evening, decrease late evening mobile phone use, reduce noise, maintain normal light-dark cycles, use mask and earplugs, minimize nightime assessment, brief relaxation or mindfulness exercise before sleep
	Pharmacological	Acute anxiety–alprazolam 0.125-0.25 mg tds, lorazepam 0.5-1 mg tds, gabapentin 100-300 mg tds, hydroxyzine 25-50 mg tds, haloperidol 0.5-1 mg tds, olanzapine 2.5-5 mg tds, quetiapine 25-50 mg tds Depression–fluvoxamine 50 mg on and titrate (max 100 mg tds) Insomnia–lorazepam 0.5-1 mg on, temazepam 15 mg on, zolpidem 5-10 mg on, zopiclone 7.5 mg on, trazodone 25-100 mg on, melatonin 5-10 mg on
Spiritual distress	Spiritual care	Spiritual assessment with FICA, therapeutic presence, treat every patient as a person, whole-person care, telechaplaincy
Open and sensitive communication		
Communicating medical information	Tele-**SPIKES** through phone or video call: **S**etting–address patient by their names, introduce oneself and explain purpose of call **P**erception–clarify understanding of disease and prognosis **I**nvitation–ask what patient wants to know and how much patient wants to know **K**nowledge–explain according to patient’s pace (disease, organs, complications, prognosis) **E**motions–empathy (the 4As: aware, allow, acknowledge, always give hope) **S**trategies–discuss parallel planning if patient is ready	
Parallel planning	Current treatment plan and advance care plan using **GOOD** framework: **G**oals–clarify goals of care, explore what is important **O**ptions–clarify treatment options, benefits vs harms, explain risk of rapid worsening **O**pinions–clarify current and advance treatment preferences, discuss plan for regular updates, identify family member to contact for regular updates **D**ocumentation–document the discussion	
Communicating through PPE	**ABC**: **a**ttend mindfully, **b**ehave calmly, **c**ommunicate clearly	
Caring for the whole team		
Exposure-related team care	Reducing contact: maximizing telemedicine, avoid unnecessary contact Mindful contact: mindful hand hygiene, mindful donning and doffing PPE	
Non-exposure-related team care	Psychosocial support	Psychological first aid (PFA)
	Self-care	**R.E.S.T**–rest, relax and set up daily routine; eat and exercise regularly; sleep, social distance and seek help if necessary; having “me time,” “we time,” “spiritual time,” reducing unpleasant “screen time” **Two-breath exercise** Breathing in, I calm down. Breathing out, I smile. Breathing in, present moment. Breathing out, wonderful moment.

## COVID-19

COVID-19 is a disease caused by the Severe Acute Respiratory Syndrome Coronavirus 2 (SARS-CoV-2).^7^ The clinical spectrum of COVID-19 can be divided into 5 categories: asymptomatic, mild disease with flu-like symptoms, moderate disease with pneumonia, severe disease with pneumonia and hypoxia, and critical disease with multi-organ failure.^8^ The hallmarks of COVID-19 include a viral phase during the first week of symptoms, followed by a pro-thrombotic hyper-inflammatory phase during subsequent weeks.^9^ Theoretically, antivirals such as remdesivir and favipiravir target the viral phase; while anti-inflammatory drugs such as dexamethasone, tocilizumab and baricitinib target the hyper-inflammatory phase.^10^ Nevertheless, only dexamethasone has been found to reduce mortality for severe and critical COVID-19 patients.^11^ Since evidence-based antiviral therapies remain lacking, supportive care has been the mainstay of COVID-19 management.

## Suffering

COVID-19 patients experience physical suffering such as fever (78%), dry cough (58%), fatigue (31%), productive cough (25%), hyposmia (25%), dyspnea (23%), myalgia (17%), headache (13%), sore throat (12%), arthralgia (11%), confusion (11%), diarrhea (10%), rhinorrhea (8%), nausea (6%), vomiting (4%), and hypogeusia (4%).^12^ For patients dying with COVID-19, dyspnea (67%) is the most prevalent symptom, followed by agitation (43%), cough (40%), drowsiness (36%), delirium (24%), pain (23%) and secretions (11%).^4^ Thirty percent of hypoxic COVID-19 patients did not complain dyspnea. They may feel calm and awake, a condition called “silent hypoxia,” but subsequently deteriorate rapidly without warning leading to death.^13,14^

COVID-19 patients have a high prevalence of psychological suffering. This includes anxiety (47%), depression (45%), sleep disturbances (34%), suicidal thoughts (23%), and post-traumatic stress disorder (13%), all of which are higher than the general population.^15-17^ Other psychosocial and spiritual suffering include fear of deterioration, fear of death, fear of dying alone, fear of transmitting the disease to family members, frightening information from social media, fear being stigmatized, worry about family members, social isolation, annoying sympathies from others, and limited communication from medical staff.^18,19^

Family members of COVID-19 patients may experience stress, anxiety, guilt, regret or anger after learning about the patient’s diagnosis. Other family suffering includes worry about patients, stress due to uncertainty, worry about contracting the virus themselves, feeling powerless, struggling to adjust their family structure without the patient at home, limited information from healthcare providers, confusing information from the media, witnessing suffering of the patient through video calls.^20^ Family members grieve about not being able to visit and help the patient, not being able to see, touch, or hold the patient, not being able to say goodbye, and not being able to have a “normal” funeral for patients.^21^

The prevalence of stress, anxiety and depression in healthcare workers (HCWs) caring for COVID-19 patients is 45%, 26%, and 24% respectively.^22^ Psychosocial suffering for HCWs include adapting to a new working environment, finding ways to work together with members from different specialties, heavy workloads, insufficient staff, fear of being infected, fear of transmitting the virus to family, feeling powerless when patients deteriorate, grief when patients die and witnessing mass casualties.^23^ Personal protective equipment (PPE)-related issues include long shifts without toilet breaks, feeling sweaty, breathless, chest discomfort and movement limitation for many hours wearing PPE, blurred eyesight with goggles, insufficient PPE, and communication challenges with PPE.^23^ Ten percent of COVID-19 patients were HCWs, though the severity (9.9% versus 29.4%) and mortality (0.3% versus 2.3%) were lower compared to all COVID-19 patients.^24^

## The Role of Palliative Care in COVID-19

Palliative care should be integrated in COVID-19 management.^10^ Basic palliative care, including the relief of physical symptoms and the provision of psychosocial support, should be practiced by all HCWs caring for COVID-19 patients.^10^ Here we outline 3 principles of palliative care that can serve as a guide for **ALL HEALTHCARE WORKERS** caring for COVID-19 patients: good symptom control, open and sensitive communication, and caring for the whole team. COVID-19 patients should have access to specialist palliative care services when suffering is unrelieved.^25^

### Good Symptom Control

#### Dyspnea

Although dyspnea is the most prevalent symptom in dying COVID-19 patients, the majority of patients did not receive medications or palliative consultation for symptom management.^26^ The WHO COVID-19 management guideline recommended the use of opioids for relief of dyspnea that is refractory to treatment of the underlying cause, such as oxygen therapy, respiratory support and corticosteroids.^10^ Although the antidyspnea effect of opioids is well-known, many patients with dyspnea did not receive opioids for their symptomatic management.^27^ Oral and parenteral opioids, but not nebulized opioids, have been shown to reduce the sensation of dyspnea without any deleterious effect on oxygen saturation.^28,29^

Oral dihydrocodeine can be initiated for exertional or resting dyspnea.^30-33^ Oral promethazine can be an alternative or add-on.^34^ Failing which, oral morphine can be prescribed. Oral antiemetic such as metoclopramide and oral laxative such as senna should be added to the regimen to prevent nausea and constipation. Specialist palliative care input should be sought if the above measures fail, for patients who are opioid-tolerant, and for patients with renal or liver failure. For post-COVID chronic dyspnea, oral mirtazapine can be prescribed.^35^

In dyspneic patients dying from COVID-19, early administration of subcutaneous opioid is recommended.^3^ An algorithm that provides a stepwise approach for opioid dosing and titration for dyspnea management has been proposed.^36^ For dyspneic patients with terminal agitation, subcutaneous midazolam can be added. Analgosedation, a strategy that manages discomfort initially with opioids such as morphine, fentanyl, hydromorphone, remifentanil, sufentanil or alfentanil before providing sedative therapy such as propofol, dexmedetomidine, ketamine, midazolam or lorazepam is preferred to standard sedative-hypnotic regimens for ventilated ICU COVID-19 patients experiencing agitation.^37,38^

The use of opioids and sedatives in dying patients is not associated with shortened survival.^39,40^ Nevertheless, since imminently dying patients tend to experience reduced consciousness that could be worsened with opioid or sedatives, the goals of treatment should be clarified with patients or their loved ones, taking into account the delicate balance between dyspnea relief and maintenance of consciousness.^41^

#### Cough

The National Institute for Health and Care Excellence (NICE) 2020 guideline recommended codeine linctus or codeine phosphate tablets as the first choice in the pharmacological management of distressing COVID-19 cough; and oral morphine as second choice.^42^ Tiotropium can be useful for post-COVID cough.^43^ Gabapentin or pregabalin can be considered for refractory post-COVID cough.^44,45^ N-acetylcysteine can be prescribed in productive cough with viscous secretions.

#### Fever

When fever is associated with other distressing symptoms such as headache or body ache, oral paracetamol can be taken if there is no contraindication. Oral NSAIDs such as ibuprofen can be another option. NSAIDs use in COVID-19 is not associated with increased mortality or poorer outcomes.^46,47^ For dying COVID-19 patients with fever, subcutaneous diclofenac sodium can be prescribed.^48^

#### Anxiety, depression, sleep disturbances and spiritual suffering

Relaxation exercises, breathing exercises and online psychological interventions can be delivered to improve the psychological outcomes of COVID-19 patients.^49-51^ Oral benzodiazepines such as alprazolam or lorazepam should be used with caution for distressing anxiety refractory to usual psychological interventions. They should be avoided in the elderly, but when required use in very low doses and taper quickly.^52^ They are best avoided for patients with acute respiratory distress.^10^ Second-line options for anxiolysis include gabapentin, hydroxyzine, haloperidol, olanzapine and quetiapine.^53^

Although non-drug interventions are preferred for situational depression, recent data suggest that antidepressant use was associated with a 40% lower risk of intubation and death in hospitalized COVID-19 patients.^54^ Selective serotonin reuptake inhibitors (SSRIs) offer the strongest protection, particularly fluoxetine, which reduced intubation and death by 70%.^54^ Fluvoxamine given during mild COVID-19 illness prevented clinical deterioration and decreased the severity of the disease.^55^

Benzodiazepines such as oral lorazepam or temazepam, and Z-drugs such as zolpidem or zopiclone, should only be used in mild to moderate COVID-19 patients with distressing sleep disturbances that have failed non-drug measures, and in the absence of respiratory distress.^56^ Clinicians should have a plan to monitor patients closely and discontinue the medications if respiration becomes compromised. Oral trazodone can be a useful alternative when respiratory compromise is a concern.^57^ Oral melatonin is another option for severe to critical COVID-19 patients with sleep disturbances.^58^

Spiritual suffering is often neglected in COVID-19 care. COVID-19 patients can experience existential loneliness, fear of death, and isolation. FICA can be used to explore spiritual concerns–F: How has COVID-19 challenged your *faith*? I: How *important* is your spiritual belief when you are affected by COVID-19? C: Have you found a way to connect with your spiritual *community*? A: Do you have any suggestion about how we can best support you and *address* your spiritual needs? To relieve spiritual suffering, we have to offer our therapeutic presence virtually or through PPE, we should treat every COVID-19 patient as a person, and provide them access to telechaplaincy if necessary.^59^

### Open and Sensitive Communication

Good physician-patient communication is instrumental in the relief of psychosocial and spiritual suffering of COVID-19 patients. WHO recommended the provision of psychosocial support in the form of exploring COVID-19 patients’ needs and concerns around the diagnosis, prognosis, and other psychosocial issues via careful listening, and addressing them by giving accurate information on their condition and treatment plans, helping them with decision-making, and connecting them with their loved ones and social support.^10^ The most effective way to relieve fear and panic of COVID-19 patients and their families is to provide them with timely and accurate medical information.^60^ Communicating openly and honestly about what is known and sharing uncertainty in a clear, consistent and specific manner while acknowledging emotional responses were suggested as important elements in effective communication during the COVID-19 pandemic.^61^

#### Communicating medical information

The provision of accurate medical information is essential in COVID-19 psychosocial care delivery. VitalTalk resource created a “COVID ready communication playbook” to assist clinicians on communicating effectively with COVID-19 patients and families.^62^ To minimize the risk of disease transmission, such communication should be conducted via telemedicine. Steps from the SPIKES protocol for breaking bad news can be adapted for communication via telemedicine.^63,64^ It can be called “tele-SPIKES.” We suggest the following steps for “tele-SPIKES”: (1) Setting–select a suitable platform on which to converse, find a quiet place, breathe and settle down, then phone or video call the patient, introduce oneself and explain purpose of the call, (2) Perception–ask how they are feeling and clarify their understanding of the disease and prognosis, listen carefully without interrupting, (3) Invitation–ask the patient what they want to know further and how much they want to know, (4) Knowledge–speak in a clear and calm manner, communicate medical information in jargon free language at the patient’s pace, explain the diagnosis and prognosis in small chunks, clarify the understanding of the medical information provided, (5) Emotions–the 4 “A”s–be *aware* of patient’s emotions through their voices and paralanguage throughout the conversation, pause and *allow* patient to express their feelings whenever necessary, *acknowledge*, validate or normalize their feelings when appropriate, *always* give hope, and (6) Strategies–discuss parallel plan: current treatment plan and advance care plan when the patient is ready, provide a plan on regular progress updates.

#### Parallel planning

As COVID-19 patients may deteriorate rapidly, there is a need for parallel planning: hoping for the best but planning for the worst.^65^ It is important for HCWs to discuss parallel planning early and openly with severe COVID-19 patients at the time of hospitalization. The GOOD framework can be a concise guide for such discussion.^66^ We suggest the following adaptations of the GOOD framework: (1) Goals–discuss goals of care, ask about expectations, explore values and ask what is important, (2) Options–clarify current treatment options, explain benefits and harms, communicate risk of rapid worsening, clarify the direction of care and treatment preferences if deterioration occurs, acknowledge prognostic uncertainty (3) Opinions–provide a clear recommendation on the current and advance treatment options, clarify treatment preferences such as ICU admission and ventilation, listen to the patient’s opinions, and try to achieve a consensus, give a clear plan if no consensus is reached, identify the family member to contact for regular updates and (4) Documentation–document the discussion on goals, options, opinions, preferences and care plan.

#### Communicating through PPE

The use of telemedicine in communicating with COVID-19 patients and their families should be maximized to reduce the risk of disease transmission. At times, communicating through PPE is still necessary. Wearing PPE can muffle a clinician’s voice and obscure facial expressions, rendering communication through PPE challenging. Brief training in mindful communication based on an ABC mnemonic to improve communication through PPE could be used to overcome these challenges.^67^ The ABC mnemonic refers to (1) Attend mindfully–breathe in and out to center oneself prior to a visit, write names or display a portrait photo on the PPE to humanize the encounter, reflect on communication asymmetries such as expert-layperson, healthy-sick, independent-dependent, cognitively healthy-cognitively impaired and so on, be aware of one’s characteristic gestures and body language, and align them with the verbal messages; (2) Behave calmly–approach patient from the front, respect the patient’s personal space, bring oneself down to eye level and project a calm attitude, avoid body language that shows frustration, anger or impatience; and (3) Communicate clearly–introduce oneself and address patients by their names, use short, simple sentences and underline one’s words with gestures if necessary, make use of pauses and speak gently and slowly, and try mirroring the patient’s mood or tone to help them feel understood.^68^

### Caring for the Whole Team

#### Exposure-related team care—reducing contact

HCWs have at least a threefold increased risk of contracting COVID-19 compared to the general population.^69^ Although the severity and mortality of COVID-19 are lower among HCWs, it is still unacceptable for HCWs to die because of their occupation.^24^ As of May 2021, at least 115,000 HCWs have died from COVID-19.^70^ To fight this pandemic, every effort needs to be made to ensure maximal safety of the HCWs caring for COVID-19 patients. To achieve this, the COVID-19 team needs to reduce its contact with COVID-19 patients through maximizing the use of telemedicine. Organizational management must invest in software, hardware and training of HCWs in the use of telemedicine.^71^ Telemedicine should be utilized for clerking patients, updating patients, meeting family members and interprofessional communication. Virtual ward rounds, clinics, family meetings, and family visits should become the norm rather than the exception. Access to fast, stable internet connectivity with secured end-to-end encryption is crucial.^71^ Remote monitoring or self-monitoring should be used to observe stable COVID-19 patients. An automated COVID-19 symptom monitoring systems should be developed to provide self-monitoring of patients at home.^72^ Robots should be deployed to deliver medications and meals in isolation wards. Drones could also be used to deliver medical supplies for home patients.^73^

To further reduce contact, the 80/20 rule, also known as the Pareto’s principle, can be very relevant.^74^ In medicine, it means 20% of medical care delivers 80% of the results. It is time to cut down the 80% of the ward rounds, blood investigations and medical care that does not contribute to the well-being of care recipients. With respect to dying patients, evidence from a systematic review indicates that around one third of patients near the end-of-life received non-beneficial treatments such as ICU admission, dialysis, transfusions, antibiotics, unnecessary blood investigations, life support and resuscitation.^75^ While a certain level of non-beneficial treatments is inevitable, HCWs need to be more mindful about the negative impact of non-beneficial treatments and the risk of disease transmission during the pandemic, particularly in those for conservative management.

#### Exposure-related team care—mindful contact

Above all, HCWs should get vaccinated for protection. For necessary contact, ideally all consultations should be pre-planned with a clear purpose. Clinicians should go through the medical records to review the history, progress, vital signs, blood investigations, and imaging prior to each consultation. Telemedicine should be considered first outside the isolation rooms remotely to identify relevant issues that require attention. PPE is of paramount importance during contact. The choice of PPE should be based on risk of exposure and possible modes of transmission.^76^ HCWs must have formal training in the use of PPE and should be supervised by a trained colleague during donning and doffing.^77^

Donning and doffing PPE requires meticulous attention to reduce contamination of HCWs caring for COVID-19 patients. In one observational study, 39% of HCWs made multiple doffing errors following patient interaction. These errors can lead to serious consequences.^78^ Mindful donning and doffing can be practiced to reduce such errors, and allay fears. We suggest the following steps (1) Mindful breathing–become aware of our in and out-breath to come back to the present moment, (2) Mindful hand hygiene–turn on the tap and let the water flow over our hands, wash the different parts of our hands and fingers so that we can use them to take care of our patients, alternatively, alcohol rub may be used, (3) Mindful donning–put on the PPE bottom-up, beginning with the shoe covers, the gown or coverall, the mask or N95 respirator, goggles, face shield, head cover, and finally the gloves; as we put on our protective gear one by one in proper sequence, we reflect on the benefits they offer us; we feel grateful for the fact that they have been proven effective and we feel thankful to the essential workers who produced them, (4) Entering–make a silent wish to speak words that bring comfort and deliver care that brings relief to the patient, then enter the room, (5) Mindful doffing–remove the PPE from the periphery to central, starting from the contaminated shoe covers, followed by the gloves, the gown or coverall, the head cover, face shield, goggles, and finally the mask or N95 respirator, imagine we are freeing ourselves from all the contamination when we remove them one by one, and (6) Mindful hand hygiene–perform hand hygiene after removing each of the contaminated pieces of PPE, then breathe in and out, relax and come back to the present moment.

#### Non-exposure-related team care—psychosocial support

Basic mental health and psychosocial support (MHPSS), a composite term used to describe any type of support that aims to promote psychosocial well-being or treat mental health conditions, should be provided to all HCWs caring for COVID-19 patients. A combined approach consisting of organizational interventions and targeted individual psychological support or specialized services should be put in place to alleviate the psychological impact of the pandemic on HCWs.^79^ Proactive organizational approaches include ensuring adequate PPEs, adequate rest, clear communication and guidelines, rapid access to occupational health and safety teams, accommodation for high-risk HCWs, support for children’s needs, regular screening of HCWs’ mental well-being as well as creating a framework to offer psychological first aid and specialized services toward HCWs needing further help.^80,81^

Psychological first aid (PFA) is a “humane, supportive response to a fellow human being who is suffering and may need support.”^82^ PFA, based on the principles of “look, listen, and link,” has been devised as a first-line psychosocial support that could be provided by all, not just by mental health professionals. This allows mobilization of societal resources and fellow HCWs working in a similar setting. HCWs could provide PFA to each other, an intervention that may be helpful for those working in restricted locations such as within the COVID-19 wards. Nonetheless, basic training in PFA is warranted to educate the providers regarding its basic principles to ensure effectiveness and reduce the risk of any adverse outcomes.^83^ To maintain the safety of PFA providers and receivers, remote administration of PFA (rPFA) through various online platforms such as the “Whatsapp” application, video calls or telephone hotlines is recommended to reach out to consenting HCWs.^84,85^

PFA providers promote safety, calmness, connectedness, and hope by actively looking out for distressed HCWs, listening to their concerns, linking them to the appropriate resources available, as well as aiding them to mobilize their own support systems. PFA providers should identify and refer HCWs to mental health services, particularly if they are show warning signs such as possible harm to self or others, have long-lasting or severe distress, or are unable to function in daily life.^83^ For HCWs that do not prefer an individualized-based approach, online psychoeducational videos, self-help materials, and webinars are also helpful.

#### Non-exposure-related team care—self-care

To complement the above support strategies, a self-care approach is equally important to promote psychological well-being and resilience. As there are many types of self-care practices, we propose an easy-to-remember self-care mnemonic R.E.S.T, which stands for (1) R–rest, relax and set up a daily routine, (2) E–eat healthy, well-balanced meals, and exercise regularly, (3) S–sleep well, stay away from the crowd and avoid physical gathering, and seek help when necessary, and (4) T–having “me time” by engaging in pleasurable activities and hobbies, having “we time” by staying connected with family and friends through online platforms, having “spiritual time” by engaging in meaningful or religious activities, and having less “screen time” checking news that can be worrying or upsetting.

For exercises, mental exercises such as practicing mindfulness or gratitude are equally important. A systematic review concluded that brief mindfulness exercises lasting 5-20 minutes daily can be effective in improving HCWs’ well-being and decreasing levels of stress and anxiety.^86^ Aside from regular mindfulness exercises at home and during work activities such as hand hygiene, a rapid two-breath mindfulness exercise can be applied to come back to the present moment. This exercise is based on a mindfulness verse by the Vietnamese mindfulness master Thich Nhat Hanh.^87^ It can be repeated silently in our mind during time of stress. The verse is as follows: Breathing in, I calm down. Breathing out, I smile. Breathing in, I come back to the present moment. Breathing out, this is a wonderful moment.

## Conclusion

The pandemic has brought immense suffering, fear and death to people everywhere. The knowledge, skills and experiences from palliative care could be used to relieve the suffering of COVID-19 patients and the people caring for them. As HCWs, we have been deemed essential workers in this pandemic. While there are certainly many more professions which are essential, it has reassured many of us to know that our efforts means so much to many people. Despite the ongoing challenges and pressures, there is also the hope that this pandemic can lead us to live more mindfully and regain a sense of doing deeply meaningful work.
